# Cerebro-Spinal Meningitis

**Published:** 1866-09

**Authors:** Juriah Harriss

**Affiliations:** Prof. Physiology in the Savannah Medical College


*Cerebro-Spinal Meningitis.* By JURIAH HARRISS, M. D., Prof. Physiology in the Savannah Medical College.

The prevalence of this disease within the past few years, in Europe and America, especially in the latter country, has attracted the attention of the profession on both continents. Numerous contributions to medical literature on this affection, and able articles in elucidation of its causes, pathology and treatment have been published by men of ability and reputation. Among the writers there is considerable diversity of opinion. Science must advance by the clash of intellects, and differences in opinions and interpretations of observations must ultimately result in truth.

The conclusion to which many recent writers have arrived is, that there is an identity of causes, as well as nature, of Cerebro-Spinal Meningitis, and Typhus or Spotted Fever. From my observations and reading, I am forced to the belief that they are distinct diseases.

It is now well demonstrated that but few causes can induce tonic spasms of the voluntary muscles. In Cerebro-Spinal Meningitis this form of muscular contraction is *almost constant*—rare in Typhus Fever. It is well known that strychnine, or irritation, or inflammation of the spinal centre, or, lastly, of the spinal nerves at their periphery, or along their course, will produce such a result. The question, then, resolves itself into this: Can a poison, such as induces Typhus Fever, one of the lowest grades of fever known, produce this irritation or inflammation of Spinal Meninges or spinal cord, which will bring about this tonic contraction of the voluntary muscles? This result, if it occurs, must be incidental, not the rule; an exception, not a constant symptom. Whilst I have been fortunate in not meeting with a very large number of cases of Cerebro-Spinal Meningitis, I have had an opportunity to note numerous cases of Typhus. From these observations, and consultation with authors, I must confess my inability to recognize anything more than a mere coincidence, when in the progress of Typhus we meet with Cerebro-Spinal Meningeal inflammation. The converse I conceive to be true, viz., that in Cerebro-Spinal Meningeal inflammation there generally exists an inflammation with an effusion of plastic lymph, whether the patient succumbs or survives. If the patient recovers, the course of the disease is not that which the Typhus presents, though protracted, nor does the peculiar rash present itself, save perhaps as an exception. Condie, in his note on Cerebro-Spinal Meningitis in Watson's work, refers to no relationship between the two diseases. Wood, in his article, "Typhus Fever," remarks: "While a student of medicine, and some years after beginning to practice, I had many opportunities of seeing the proper Typhus Fever, which prevailed epidemically in different parts of the United States, beginning in New England so early as 1807, reaching our own neighborhood in the Winter of 1812–13, and continuing to lurk in the lanes and alleys, among the crowded poor of Philadelphia, until the year 1820 or 1821, when it disappeared," etc. In 1836 he witnessed another epidemic. He makes no mention of the identity of the two diseases. Mark that it prevailed in *Winter* and *in a cold climate in lanes and alleys.*

Under the head of "Anatomical Characters" he says: "Though various evidences of deranged structure are presented, upon dissection after death, in the advanced stages of Typhus, *yet there is no one characteristic and essential lesion*, unless, perhaps, the state of blood and the petechial eruption. All the others may be considered *incidental.*" No reference is made to opisthotonos. So far as I am informed M. Andral, whom no medical observer in his day excelled as a medical philosopher, recorded, with his usual accuracy, the first case, in 1821, of a female who died of Cerebro-Spinal Meningitis. The post mortem exhibited the false membrane at base of cerebellum, and over the medulla oblongata, between the arachnoid and piamater. Ollivier and Abercrombie subsequently described the same. M. Mallet believed that there was an intermission in the fever, and Gassaud regarded Cerebro-Spinal Meningitis to be of malarial origin. I quote from the same authority the testimony of Dr. Mayne, in connection with an epidemic which occurred in Ireland in 1846: "The *serous membrane covering the brain and spinal marrow was invariably found to be the seat of extensive inflammation, and unlike the more ordinary forms of arachnitis, the spinal arachnoid always suffered much more severely than the cerebral.*"

In the *London Lancet*, Sept., 1818, there is a concise and accurate description of an epidemic, by Dr. Sanderson, which prevailed in Dantzic, to which point he was sent by his government, to investigate the "nature, causes, prevention and treatment of the disease, as well as the rise, progress and extent of the epidemic." From his investigations he arrived at the same conclusion that Gassaud had reached, that the disease prevailed as an epidemic in malarial districts—"*Contraction of the muscles of the back of the neck is the symptom which had been regarded as the most characteristic of the disease.*" In this report he however contends that the rigidity of the muscles of the neck was not tetanic. "The head was thrown backwards, and the patient complained of agonizing pain in the nape and occiput." The body could be readily bent forward by assistance, but the chin could not be brought against the chest. He mentions but one case accompanied by a petechial eruption, and *that made its appearance on the face.*

Another important fact, the result of his observations at Dantzic, is that he was led to the conclusion that, unlike Typhus Fever, the *Cerebro-Spinal Meningitis was not contagious.*

In objecting to the views of those who advocate the identity of Typhus or Spotted Fever and Cerebro-Spinal Meningitis, I urge first, that Typhus, with its slow insidious progress, low grade of fever and characteristic eruption, has been closely studied for thirty-five years; the causes carefully observed, and the pathological changes scrupulously noted, by most skillful observers. No claim, as to identity, was advanced until a comparatively recent date. Secondly, if there was an identity, the local manifestation of *eruption* in the one, and *effusion of plastic lymph* and opisthotonos in the other, should have been more frequently noticed, to say nothing of rapid death attending Cerebro-Spinal Meningitis. These two arguments pertain to negative or exclusive reasoning. I will next proceed to the more positive: 1st, as to contagion—It cannot, with any semblance of truth, be denied that all authorities admit the contagiousness of Typhus Fever. I know of no authorities who believe that Cerebro-Spinal Meningitis is contagious. Its contagiousness cannot be sustained by facts. It is an accepted proposition, unless I have been badly instructed by authors, and have made false observations, that Typhus or Spotted Fever prevails in jails, on shipboard, or in crowded, damp, and narrow streets; among a population who breathe impure air, with a limited supply of food, aud that of inferior quality. Does epidemic Cerebro-Spinal Meningitis peculiarly infest these localities, or exist alone in such impure atmospheres? Such has not been the record. Upon the contrary, Cerebro-Spinal Meningitis mostly prevails in miasmatic regions, (see Drake), attacking suddenly persons in full health, those who enjoy a country atmosphere, free exercise, abundant nutritive food, and where there is no crowding of individuals in confined limits. Dr. Watson says that "these pathologists are in error who maintain that the essence of contin ued fever (in which he includes Typhus) is inflammation of the brain; we fail to discover, in many instances, any traces of inflammation, upon inspecting the dead brain." In his opinion, then, the disturbance of the brain, during life in Typhus, as in Typhoid is functional, not organic or inflammatory.

Dr. De Costa in the *American Journal Medical Sciences*, January, 1866, gives an accurate account of the Typhus Fever, which prevailed as an epidemic, in Philadelphia, in the Winter and Spring of 1864. Under the division of anatomical lesions, he says: "In no instance was any inflammation of the brain discovered, nor did the brain substance itself look other than healthy. There was no effusion into the ventricles, nor was even congestion of the membranes a constant feature; in only one instance were the veins on the surface filled with dark blood." No reference was made by him, though he availed himself of abundant opportunities to make post mortem examinations, to the *peculiar* symptoms of opisthotonos, or rigidity of muscles of the posterior cervical region, nor to the existence of an effusion of plastic lymph on the base of cerebellum and over medulla oblongata.

Dr. S. F. Prewett, *St. Louis Medical and Surgical Journal*, 1865, states that in an epidemic which prevailed in Missouri, in 1861–62, he had occasion to observe a large number of cases. He records but one case which presented anything like petechial eruption.

In the *London Lancet*, as late as June, 1865, a number of cases of Typhus are narrated, and no reference made to a cerebro-spinal complication. "The researches of John Reid, and all subsequent observers (I quote from Dr. Murchison, of London,) have demonstrated that there exists no relation whatever between the degree of vascularity and the amount of subarachnoid fluid on the one hand, and the severity of the cerebral symptoms on the other. It is now universally admitted among pathologists that the lesions of Typhus are quite independent of inflammatory action." This is the conclusion to which I have arrived from observation of cases. The investigations of John Reid, Peacock, Jenner, Jacknot, Barrillin, and almost all modern observers who have had much experience in the two diseases, and enjoyed the opportunities of making post mortem examinations, lend force to my conviction. M. Molring, of the Prussian army, examined the cerebral membranes and subarachnoid serosity in upwards of two hundred cases of Typhus. In instance could he detect a pus or exudation corpuscle. The Prussian Surgeon adds: "Still it is not surprising that many modern practitioners, having little experience in post mortem examinations, regard the cerebral lesions of Typhus as due to inflammation, and that when the disease shows itself in countries where it is little known this should be the common opinion, with petechial eruption, accompanied with inflammation and effusion of plastic lymph on surface of the brain." In his extensive experience, he records but two cases of petechial eruption, associated with inflammation and effusion of plastic lymph on surface of the brain. In these two he does not mention that the lymph was found on what I consider the "point d'election," in most cases of Cerebro-Spinal Meningitis. The locality of the disease, as it most frequently presents itself, is most commonly at base of cerebellum, and over the medulla oblongata.—*Sav. Jour. Med.*

